# Simultaneous Semantic Segmentation and Depth Completion with Constraint of Boundary

**DOI:** 10.3390/s20030635

**Published:** 2020-01-23

**Authors:** Nan Zou, Zhiyu Xiang, Yiman Chen, Shuya Chen, Chengyu Qiao

**Affiliations:** 1 College of Information Science and Electronic Engineering, Zhejiang University, Hangzhou 310027, China; znmax@zju.edu.cn (N.Z.); chenyiman@zju.edu.cn (Y.C.); shuya_chen@zju.edu.cn (S.C.); 3140104437@zju.edu.cn (C.Q.); 2 Zhejiang Provincial Key Laboratory of Information Processing, Communication and Networking, Zhejiang University, Hangzhou 310000, China

**Keywords:** CNN, semantic segmentation, depth completion, multi-task learning

## Abstract

As the core task of scene understanding, semantic segmentation and depth completion play a vital role in lots of applications such as robot navigation, AR/VR and autonomous driving. They are responsible for parsing scenes from the angle of semantics and geometry, respectively. While great progress has been made in both tasks through deep learning technologies, few works have been done on building a joint model by deeply exploring the inner relationship of the above tasks. In this paper, semantic segmentation and depth completion are jointly considered under a multi-task learning framework. By sharing a common encoder part and introducing boundary features as inner constraints in the decoder part, the two tasks can properly share the required information from each other. An extra boundary detection sub-task is responsible for providing the boundary features and constructing cross-task joint loss functions for network training. The entire network is implemented end-to-end and evaluated with both RGB and sparse depth input. Experiments conducted on synthesized and real scene datasets show that our proposed multi-task CNN model can effectively improve the performance of every single task.

## 1. Introduction

Scene understanding [1,2,3] is an essential task in lots of intelligent applications such as robotics navigation, AR/VR and autonomous driving. As the core content of scene understanding, semantic [4,5,6,7,8] and depth estimation [9,10,11,12] parse the scene in the view of semantics and geometry, respectively. In recent years, much research focusing on either depth estimation or semantic segmentation has been carried out. With the help of deep learning technologies, great success has been made.

Semantic segmentation refers to the classification and labeling of each pixel in an image, thereby dividing the image into several semantic meaningful regions, and converting the original color-data image into a pixel-level class-labeled image. Following the success of deep neural networks in image classification [13], the invention of fully convolutional neural network (FCN) [5] makes the pixel-level semantic labeling possible. Based on FCN, recent studies develop deconvolution-based architectures to improve segmentation accuracy [6,7]. Deeplab [8] proposes an atrous convolution to tackle the problem of low-resolution features caused by the traditional cascading pooling structure. Their later work [14] introduces bottleneck and skip-connection structure with Resnet [15] framework. ERFNet [16] further proposes a non-bottleneck structure to reduce computing complexity. Extra data sources can also be introduced to improve performance through feature fusion. Dense or sparse depth images, acquired with range sensors such as Kinect or Lidar, are used to strengthen the semantic segmentation results [17,18]. A depth-aware CNN [19] is proposed to optimize the convolution operation with the sparse depth input. To further improve the segmentation results on small objects or near the borderline among classes, boundary-aware convolution is proposed in our previous work [20].

Depth completion refers to predicting dense depth information for each pixel given the sparse depth input. A closely related area is depth estimation from a single RGB image. Lots of deep learning based algorithms are proposed for depth estimation from one image [21,22], and they usually suffer from a similar over-fitting outcome since the problem itself is ill-posed. In contrast, depth completion [12,23] is able to achieve better performance than depth estimation from RGB since it can have sparse depth points as references. IP-Basic [23] uses some delicate morphological operations, e.g., inversion, dilation and hole closure, to perform depth completion. Following FCN’s network structure, some methods treat the depth completion as depth up-sampling work with pixel-level prediction [11]. To counter the special situation of missing data, Uhrig et al. [12] proposes a sparsity invariant convolution. It considers the irregular distribution of sparse inputs and designs the mask-based convolution operating on the valid data only.

Data fusion is a good way to improve the performance of the tasks. In recent years, studies utilizing heterogeneous data [17,24,25,26,27,28,29] for semantic or depth tasks have been carried out. Couprie [17] concatenates the RGB and depth image and builds a multi-scale semantic segmentation network with early fusion. Hazirbas proposes a middle fusion structure FuseNet [24] for better rgb-d semantic segmentation. For depth completion, image-guided methods have achieved quite a lot of attention. Mal [25] first introduces RGB and sparse depth simultaneously to perform better depth estimation. Jaritz [26] improves the work by combining RGB and depth features with late fusion in a multi-task NASNet-based [27] network. Other than introducing RGB data, researchers have also attempted to integrate other features, such as obstacle boundary and surface normal [28,29].

Joint modeling [26] multiple closely related tasks appears to be another feasible way to improve the performance of the overall mission. Conditional random fields (CRF) is usually used to solve the scene-understanding problem by multi-task joint modeling. Murphy [30] first proposes a multi-task joint model for scene classification and target detection in 2003, which effectively combines global and local feature information in image space. Semantic segmentation, which is considered relevant to other similar tasks such as object detection and classification, could be improved by multi-task learning [31,32,33,34]. A cross-stitch structure among feature layers is proposed to better learn the shared features [33]. Uhrig [34] further studies semantic and instance segmentation together based on the fully convolutional neural network. In [35], Kendall proposes to learn the weights among the loss functions for different tasks, i.e., semantic segmentation, instance segmentation and depth estimation, and achieves better performance.

In this paper, we focus on simultaneously performing semantic segmentation and depth completion. We believe the two tasks have some hidden similarities and relationships between them, although they are not obvious. A heuristic observation is that the depth distribution pattern in the same semantic regions may be similar, such as roads and walls. Furthermore, although the two tasks depend on the heterogeneous data input, i.e., color image and sparse range data respectively, there is some evidence that using both data as input can be beneficial for each task [20].

**Figure 1:**
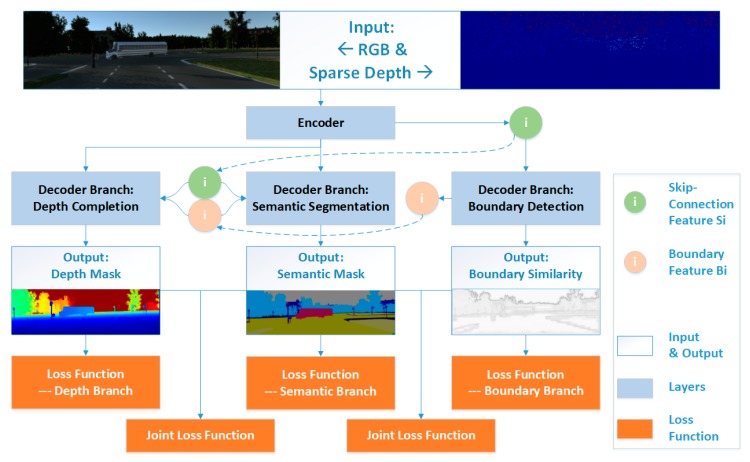
Proposed simultaneous semantic segmentation and depth completion multi-task network (SSDNet). Boundary constraint is embedded into the two major tasks via multi-scale feature sharing and cross-task joint loss function.

Building upon our previous work on boundary-aware CNN [20] for semantic segmentation, we propose a multi-task CNN for simultaneously predicting the semantic and dense depth of scenes. Besides the network model for the two major tasks, an extra boundary detection sub-net is also designed in the framework. Taking the boundary as the regularizing constraint between the two major tasks, predicted semantic and dense depths could be largely improved. The entire network adopts the single-encoder-multi-decoder architecture, which is able to extract more effective features suitable for all of the three sub-tasks during the encoding process. Skip connections are also designed among all of the separate decoders for each sub-task to strengthen the feature sharing. To the best of our knowledge, it is the first time that the semantic segmentation and depth completion tasks are jointly modeled in this close-coupled manner. The experimental results show that our end-to-end multi-task CNN is able to boost the performance of each task. The framework of the proposed simultaneous semantic segmentation and depth completion multi-task network (SSDNet) is shown in Figure 1.

The main contributions of our work are threefold. First, a triple-task network with single-encoder-multi-decoder architecture is designed for simultaneous predicting semantic and dense depth of an image. It takes RGB and sparse depth data as input, capable of utilizing the complementary information hidden in each of the heterogeneous data. Second, a boundary constraint is embedded into the two major tasks via multi-scale feature sharing and cross-task joint loss function. The boundary plays a role of bridge connecting the semantic and depth prediction tasks and strengthens the relationship between them. The last contribution is the end-to-end implementation of the entire network and evaluation of the method on different datasets. The experiments on both synthesized (Virtual KITTI [36]) and real datasets (Cityscapes [37]) demonstrate that our method can effectively improve the performance for both depth completion and semantic segmentation.

## 2. Proposed Methods

In this section, the proposed network architecture is introduced first, and the loss functions for training the network are described later.

### 2.1. Network Architecture

The overall network is based on FCN structure with a single sharing encoder and multiple branch decoder. As shown in Figure 1, the architecture of the proposed SSDNet is mainly composed of a feature-sharing encoder and three branch decoders corresponding to boundary-detection, semantic-segmentation, and depth-completion tasks, respectively.

#### 2.1.1. Feature-Sharing Encoder

**Figure 2:**
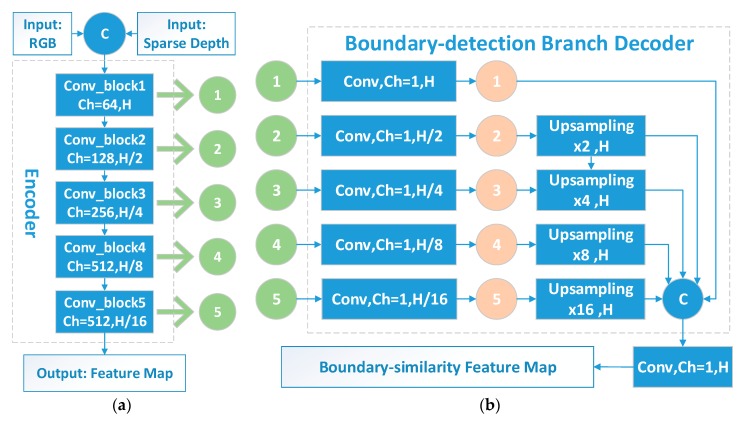
Proposed feature-sharing encoder (**a**) and boundary-detection decoder (**b**). Blue node C refers to the concatenate layer. The green and pink node with the number i represent the skipped and the boundary similarity feature from the i th scale, respectively. H represents the raw resolution of the input image and $H/2^{i-1}$ refers to the resolution after the i th pooling. Ch=m represents the output channel number m for each Conv_Block. The bilinear interpolation upsampling layer in (**b**) is denoted as Upsampling $\times 2^{i-1}$, where $2^{i-1}$ represents the upsampling ratio.

The main structure of the proposed feature-sharing encoder is based on VGG’s [13] multi-scale cascading convolutions, as shown in Figure 2a. It consists of 5 convolution blocks denoted as Conv_block. The scaled outputting features from Conv_block, marked as green nodes $S_{i}$, are transferred to all of the three subsequent decoder branches in the form of skip-connection, as shown in Figure 1.

#### 2.1.2. Decoder for Boundary Detection

The boundary is valuable information to hint the discontinuity in the semantic labels and depth image. Therefore, a boundary detection decoder is designed to produce the required boundary similarity to the other two major tasks. As shown in Figure 2b, together with the output of the encoder part, different scales of skip-connection features are also fed into the boundary-detection branch. The boundary computed from the semantic ground truth image can be utilized as supervision signals to train the boundary detection branch. Different boundary feature maps $B_{i}$ from the ith scale, marked as pink nodes in Figure 2b, are then introduced to the following semantic segmentation and depth completion branches.

#### 2.1.3. Decoder for Semantic Segmentation and Depth Completion

**Figure 3:**
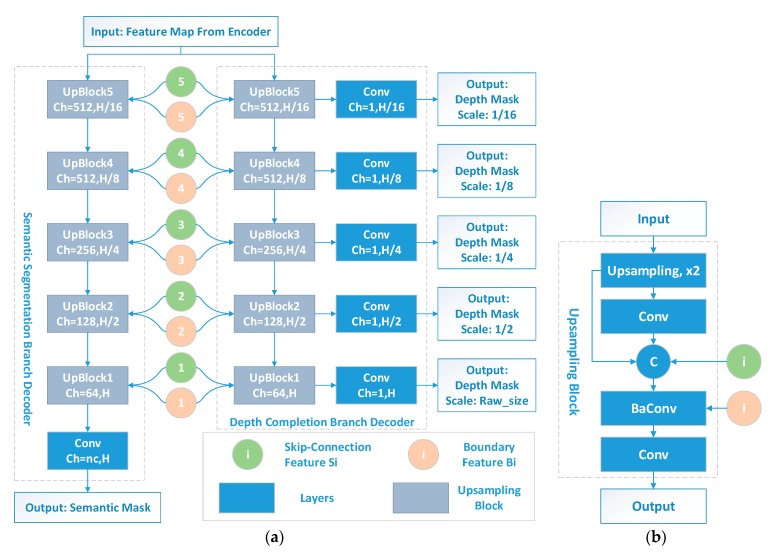
Semantic segmentation and depth completion decoder (**a**) and UpBlock structure (**b**). nc represents the number of semantic classes. BaConv refers to the boundary-aware convolution [20]. The bilinear interpolation upsampling layer is used in (**b**) as Upsampling, x2.

Besides the feature maps from the encoder, the semantic and depth decoder branches also share the multi-scale skipped features and the boundary feature maps, as shown in Figure 3a. The core modules for both branches are the multiple UpBlocks, where the boundary feature $B_{i}$ is absorbed to construct a special boundary-aware convolution layer BaConv [20], as illustrated in Figure 3b. With the guidance of the boundary, boundary-aware convolution could focus more on the regions with similar semantic features and gather the contributions more adaptively to produce the output. The operation of boundary-aware convolution is shown in Equation (1):

(1) $$y(p)=\sum_{p_{n}\in K}w(p_{n})\cdot x(p_{n})\cdot (1-B(p_{n}))$$

where the output y(·) at the position p is produced by the convolution of three parts, i.e., kernel weight w(·), input feature map x(·) and boundary similarity feature B(·). $p_{n}$ denotes each position in the local window K around the target position p. The size of K is defined by the convolution kernel size, and parameters in w(·) are determined through the training process.

Comparing with standard convolution operation, BaConv introduces the boundary-similarity term B(·) and brings the idea of adaptively setting contributions to each $p_{n}$. Based on BaConv, pixels which have higher similarities as object boundaries will have a higher weight of $B(p_{n})$ and have less impact on the convolution results.

Following five UpBlocks, a 1 × 1 convolution layer with channels equal to the class number nc is employed to produce the final semantic results. For the depth completion branch, a 1 × 1 convolution layer with an output channel number of 1 is introduced after each UpBlock, and produces a normalized depth prediction on each scale. All of the five scaled outputs are used for producing depth task loss during training, while only the depth outputs in the final scale are used in the testing stage.

### 2.2. Loss Function

Given the network model and training samples $\left\{I_{p},I_{d},G_{d},G_{s},G_{b}\right\}$, where $I_{p}$ and $I_{d}$ separately represent the RGB image and sparse depth data,$G_{d}$,$G_{s}$ and $G_{b}$ represent the ground-truth for dense depth, semantic labels and boundary, the loss functions for each single and joint task have to be designed. Boundary ground truth $G_{b}$ can be calculated from the discontinuity of the semantic ground truth. The output of the triple task is denoted as $P_{s}$, $P_{d}$ and $P_{b}$, which represent semantic, depth and boundary prediction, respectively.

#### 2.2.1. Loss Function for Depth Completion

For the depth completion branch, the model can be optimized by L1 and L2 loss between the prediction and the ground truth. SSIM loss [38] is also induced to compare the features of brightness $l\left(P_{d},G_{d}\right)$, contrast $c\left(P_{d},G_{d}\right)$, and structure $s\left(P_{d},G_{d}\right)$ between the completed depth images $P_{d}$ and the ground truth $G_{d}$. SSIM loss is defined as:

(2) Loss−SSIM(Pd,Gd)=1W∑wil(Pdwi,Gdwi)⋅c(Pdwi,Gdwi)⋅s(Pdwi,Gdwi)

(3) $$\mathrm{where}\;\begin{array}{ccc} \{\begin{array}{c} l\left(a,b\right)=\frac{2\mu_{a}\mu_{b}+C_{1}}{\mu_{a}^{2}+\mu_{b}^{2}+C_{1}}\\ c\left(a,b\right)=\frac{2\sigma_{a}\sigma_{b}+C_{2}}{\sigma_{a}^{2}+\sigma_{b}^{2}+C_{2}}\\ s\left(a,b\right)=\frac{2\sigma_{ab}+C_{3}}{\sigma_{a}\sigma_{b}+C_{3}} \end{array} & , & \{\begin{array}{c} \mu_{a}=E(a)\\ \sigma_{a}=\sqrt{E(a^{2})-E^{2}(a)}\\ \sigma_{ab}=\sqrt{E(ab)-E(a)E(b)} \end{array} \end{array}$$

In Equation (2), W represents the number of 11x11 sliding windows to calculate local image quality. SSIM loss for the overall image, i.e., $Loss_{-}SSIM$, is obtained by averaging all of the sliding windows. In Equation (3), a and b are the parameters for the metric functions, E(·) represents the expectation function to calculate the mean value for the feature map, and $\mu_{a}$, $\sigma_{a}$, $\sigma_{ab}$ denote mean, variance, covariance, respectively. $C_{1}$, $C_{2}$ and $C_{3}$ are the constants to avoid instability when the denominator is close to zero. Empirically, they are set as: $C_{1}=1e-4$, $C_{2}=9e-4$ and $C_{3}=C_{2}/2=4.5e-4$.

In summary, the final loss function for depth completion is composed of three terms:

(4) $$Loss_{d}=\alpha_{d1}*Loss_{-}L_{1}+\alpha_{d2}*Loss_{-}L_{2}+\alpha_{d3}*Loss_{-}SSIM$$

where $\alpha_{d1}$, $\alpha_{d2}$ and $\alpha_{d3}$ are the weights for each loss, respectively. Empirically, they are set as $\alpha_{d1}=\alpha_{d2}=0.4$, and $\alpha_{d3}=0.2$ in our implementation.

#### 2.2.2. Loss Function for Semantic Segmentation and Boundary Detection

For semantic segmentation-related tasks, the class weighted cross-entropy is applied in the loss function. Given $gt_{jk}$ and $pred_{jk}$ the probability of the jth pixel labeled as the kth class in the ground truth and the prediction, respectively. The class-weighted cross-entropy (WCE) is given as:

(5) $$Loss_{s}=WCE\left(G_{s},P_{s}\right)=-\sum_{j=0}^{N-1}\sum_{k=0}^{nc-1}\beta_{j}gt_{jk}\log(pred_{jk})$$

where $\beta_{j}$ represents the proportion of pixels with the semantic category j in the whole sample dataset, N and nc represent the total number of pixels and semantic categories. Class-weighted cross-entropy (WCE) is used to evaluate the output of the semantic segmentation branch decoder.

Similarly, boundary-detection could be defined as a binary semantic segmentation problem and two-class-weighted cross-entropy is employed in Equation (6):

(6) $$Loss_{b}=-\sum_{j=0}^{N-1}\left(\beta_{j}gt_{j}\log\left(B(pred_{j})\right)+\left(1-\beta_{j}\right)\left(1-gt_{j}\right)\log\left(1-B(pred_{j})\right)\right)$$

where $B(pred_{j})$ denotes the boundary-similarity of the jth pixel in the boundary-prediction map and $gt_{j}$ denotes if jth pixel is labeled as a boundary.

#### 2.2.3. Loss Function for Joint Tasks

Aiming to enhance the correlation among different tasks and further improve the overall generalization performance, joint cross-task loss functions are proposed. Boundary constraint is embedded into the two major tasks via multi-scale feature sharing and cross-task joint loss function. The boundary plays the role of bridges connecting the semantic and depth prediction tasks and strengthens the relationship between them. In brief, the positions of the semantic boundary, depth boundary and boundary predicted through the detection sub-task should all be compatible.

Specifically, defining local boundary function $f_{BS}\left(\cdot \right)$ on the semantic prediction image $P_{s}$, the semantic boundary confidence in the horizontal $f_{BS_{-}u}\left(\cdot \right)$ and vertical direction $f_{BS_{-}v}\left(\cdot \right)$ can be respectively computed as shown in Equation (7):

(7) $$\begin{array}{ccc} f_{BS_{-}u}\left(P_{s:u,v}\right)=\{\begin{array}{cc} 0, & P_{s:u,v}=P_{s:u-1,v}\\ 1, & P_{s:u,v}\neq P_{s:u-1,v} \end{array} & , & f_{BS_{-}v}\left(P_{s:u,v}\right)=\{\begin{array}{cc} 0, & P_{s:u,v}=P_{s:u,v-1}\\ 1, & P_{s:u,v}\neq P_{s:u,v-1} \end{array} \end{array}$$

where the subscript u and v represent the pixel position in the horizontal and vertical direction, respectively. Then the semantic-boundary joint loss function is defined in Equation (8). The boundary calculated from semantic prediction should have the structure-similarity with the boundary-similarity prediction result. The semantic-boundary joint loss function can be minimized when the pixels at the semantic boundary ($f_{BS}\left(\cdot \right)=1$) has a high boundary-similarity ($P_{b:u,v}$ reaches the maximum value of 1).

(8) Losssb=1N∑u,v(fBS−u(Ps:u,v)e−Pb:u,v +fBS−v(Ps:u,v)e−Pb:u,v )

For the depth prediction, the predicted $P_{d}$ is a continuous quantity and the local gradients on $P_{d}$ could be built in Equation (9). The larger the gradient is, the more the pixel tends to the position of a boundary. Numerically, the partial derivatives could be calculated in Equation (10):

(9) $$Grad_{u}\left(P_{d}\right)=\left|\partial P_{d}/\partial u\right|\;,\;Grad_{v}\left(P_{d}\right)=\left|\partial P_{d}/\partial v\right|$$

(10) $$\begin{array}{ccc} \partial P_{d:u,v}/\partial u=P_{d:u,v}-P_{d:u-1,v} & , & \partial P_{d:u,v}/\partial v \end{array}=P_{d:u,v}-P_{d:u,v-1}$$

Then the semantic-depth joint loss $Loss_{sd}$ can be formulated in Equation (11). It can reach the minimum when the pixels at the semantic boundary ($f_{BS}\left(P_{s:u,v}\right)=1$) has a high depth gradient $Grad\left(P_{d:u,v}\right)$.

(11) Losssd=1N∑u,v(fBS−u(Ps:u,v)e−Gradu(Pd:u,v) +fBS-v(Ps:u,v)e−Gradv(Pd:u,v) )

Finally, the full loss function for the entire multi-task model is defined as:

(12) $$Loss=Loss_{d}+Loss_{s}+Loss_{b}+Loss_{sb}+Loss_{sd}$$

## 3. Experimental Results

In this section, the experimental setup and the evaluation datasets are described first. Then the quantitative and qualitative results are presented with some comparisons to state-of-the-art methods.

### 3.1. Experimental Setup and Dataset Introduction

To evaluate the performance of the proposed network, we introduce metrics for semantic segmentation and depth completion task respectively. As shown in Table 1, $n_{lk}$ is the number of pixels that labeled as class l and predicted as class k, nc indicates the number of classes, and $t_{l}=\sum_{k}n_{lk}$ is the number of pixels with ground truth class l, and $N=\sum_{l}t_{l}$ represents the total number of all pixels. For depth completion metrics, $d_{j}$ and ${\hat{d}}_{j}$ represent the ground truth and depth prediction of the jth pixel.

Among semantic segmentation evaluation metrics, Acc stands for the total correct-predicted pixels in the overall image, and mAcc denotes the mean accuracy among different classes. mIoU is the average between the IoU (the ratio between the correct-predicted area and the union area of the ground truth and the predicted areas) of different semantic labels over all the images, and fwIoU represents the class-weighted IoU metric.

The network model is implemented using the PyTorch framework and trained on the NVIDIA GeForce GTX 1080 Ti with 11GB of graphics processing unit (GPU) memory. The network parameters are randomly initialized by Xavier, and the initial offset is set to zero. The loss function is optimized using the SGD optimizer in the experiment, where the initial learning rate is set to 1 × 10^−4^ and the batch size is set to 4. The experiment is done primarily on the Virtual KITTI and Cityscapes datasets.

- Virtual KITTI [36] is a synthetic outdoor dataset. The dataset contains 10 different rendering variants in each sequence, one of them is an outdoor environment cloned as close as possible to the original KITTI benchmark and the others are geometry transformed or adjusted with weather conditions from the cloned one. Each RGB image in the dataset has a corresponding depth image and semantic segmentation groundtruth. The ground truth depth maps are randomly down-sampled to only 5% of the original density to produce the sparse depth input: 11,112 images are randomly selected for training, 2320 images for validation and 3576 images for testing.
- CityScapes [37] is a real outdoor dataset, which contains high-quality semantic annotations of 5000 images collected in street scenes from 50 different cities. A total of 19 semantic labels are used for evaluation. They belong to 7 super categories: ground, construction, object, nature, sky, human, and vehicle. The ground truth of depth (disparity) is provided by the SGM method [37]. In the experiment, the original disparity images are randomly down-sampled to 5% density and used as sparse depth input. The training, validation, and testing sets contain 2975, 500 and 1525 images, respectively.

### 3.2. Experiment Analysis: Virtual KITTI

Virtual KITTI [36] is a synthetic outdoor dataset and mostly used for ablation experiments among models with different settings. All the models are trained from scratch and do not rely on any pre-training model.

#### 3.2.1. Experiments on Semantic Segmentation

The evaluation of the experiment is first carried out on the Virtual KITTI dataset. The specific settings of the comparative experimental model are listed as follows:

1. BaCNN: Baseline model proposed [20]. BaCNN is based on the backbone of FCN8S [5], and modified by replacing the first layer of each Conv_Block into the boundary-aware convolution. With the help of a front-built boundary-detection sub-network, the boundary-similarity map is introduced.
2. SSDNet_Sem: Remove the depth completion branch from our proposed multi-task learning framework. It could also be understood as two modifications to the BaCNN model:
  (a) BaCNN employs the boundary-detection sub-network as one cascading task and produces a boundary-similarity map for the following semantic segmentation sub-network, while SSDNet_Sem treats the boundary task as a dependent branch and shares the encoder features. Moreover, other than independent loss functions for boundary and semantic sub-task, SSDNet_Sem model could also be optimized by joint semantic-boundary loss function;
  (b) Comparing with BaCNN who performs Early Fusion with introducing boundary-similarity in the encoder stage, SSDNet_Sem performs Later Fusion in the decoder stage.
3. SSDNet (Full model): The complete network model with multi-tasks optimized by the full joint loss function.
4. SSDNet_ind: The complete network model without using joint loss functions.

The quantitative comparison results are shown in Table 2. For following tables, we highlight our method’s performance in bold, and underline the best performance. As expected, all of the three models perform better than the single semantic task model FCN8S [5]. With adding of the boundary and depth task, the performance is gradually improved. BaCNN [20] introduces boundary detection task and replaces the standard convolution with the boundary-aware convolution. SSDNet_Sem achieves further improvement than the baseline BaCNN model. It is due to the changes in the boundary fusion phase (from early fusion to later fusion) and the task hierarchy from the original cascaded style into a parallel multi-task architecture. By adding the depth completion task, the full SSDNet model achieved further significant improvements compared to SSDNet_Sem and performs the best on all four metrics. Without any help from cross-task joint loss functions, SSDNet_ind performs a little lower than our full model and supports the effectiveness of cross-task joint loss functions. The full model also has lower complexity and higher real-time performance than FCN8S and BaCNN (FLOPs are all computed under image resolution of 125 × 414).

**Figure 4:**
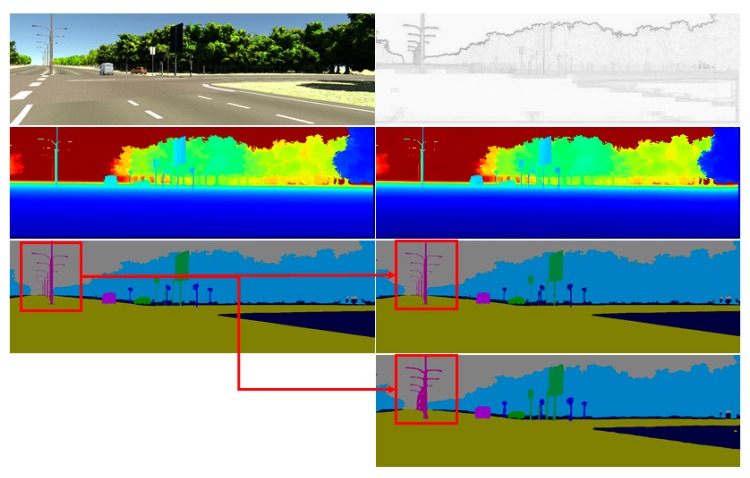
The SSDNet experimental results on Virtual KITTI. In an order of top-down: the first column shows the RGB color image, the depth ground truth and the semantic segmentation ground truth; the second column shows the boundary-similarity result, the depth completion output, the semantic segmentation result, and corresponding BaCNN [20] result respectively.

The qualitative results are shown in Figure 4, where the predictions of depth and semantics look good with the help of boundary prediction. Comparing to the baseline BaCNN model, the full multi-task model is able to produce much shaper segmentations on very close objects (as marked in the red box). This also proves that the proposed multi-task joint network can promote the performance of every single task.

#### 3.2.2. Experiments on Depth Completion

Several ablation experiments are conducted with the following model configurations:

1. SSDNet (full model): The proposed semantic segmentation and depth completion multi-task network.
2. SSDNet_Dep: Removing the semantic branch from the full model, but still using both sparse depth and RGB image as input.
3. SSDNet_Dep_d: Using sparse depth as the only data source on the model of SSDNet_Dep.
4. SSDNet_Dep_rgb: Using RGB image as the only data source and perform depth prediction on the model of SSDNet_Dep.
5. SSDNet_ind: The complete model without using joint loss functions.

In the depth completion ablation experiment, the RMSE and MAE are analyzed for depth outputs in the range of 20 m, 50 m and 100 m, respectively. Sparse depth points and image pixels become sparser with the increasing of range, leading decreasing of prediction accuracy along the distance. The experiments on these three ranges can well represent the system performance in near, middle and far range ahead of the vehicle. The quantitative comparison results are shown in Table 3, where n/a represents unpublished data. The full SSDNet performs the best among all of the models. Compared with the full model, SSDNet_Dep obtained slightly worse results on the depth prediction accuracy, which shows the importance of the semantic task to the depth completion. If only one type of data could be used, SSDNet_Dep with sparse depth can achieve better results than using the RGB image. However, they are both worse than SSDNet_Dep with full heterogeneous input, which inversely demonstrates the advantages of data fusion. SSDNet_ind is a little worse than the full model, which proves that cross-task joint loss function could help improve depth accuracy. Compared with the traditional methods such as MRF [39], TGV [40] and the state-of-the-art CNN methods such as Sparse-to-dense [25] and SparseConvNet [12], our full model also performs the best.

### 3.3. Experimental Analysis on CityScapes

To further evaluate the proposed SSDNet model in a real environment, this section conducts experiments in the CityScapes dataset and compares with state-of-the-art methods. Unlike the simulated Virtual KITTI dataset, the CityScapes dataset contains more noises in the original RGB and disparity (depth) image. The proposed SSDNet model is trained from scratch and does not rely on any ImageNet pre-training. The quantitative verification results for the semantic segmentation task are shown in Table 4, where IoU_cat and IoU_cla represent mIoU corresponding to all of the 7 categories and 19 classes, respectively; fwt represents the running time of each frame in seconds. With the help of the multi-task learning framework, the proposed SSDNet model performs better than the baseline BaCNN [20] and most of its counterparts. Compared with the state-of-the-art real-time CNN methods such as encoder-decoder based ENet model [41] and its loss-edited version [42], ESPNet [43] and two-branch-fusion Fast-SCNN [44], our full model performs better in IoU without costing more time. With fewer layers built in the network and without any pre-training on ImageNet, our method performs still slightly worse than ERFNet [16]. However, our full SSDNet model with three tasks can run at 100 fps, which is 2 times faster than ERFNet.

Detailed category evaluation comparisons with the baseline are shown in Table 5. The statistics show that our SSDNet outperforms the baseline BaCNN model in almost all categories, which verified the effectiveness of our proposed multi-task learning framework.

Compared to the dense depth map in Virtual KITTI, the depth (disparity) ground truth in the Cityscapes dataset is much noisier and only semi-dense with lots of holes in it. Our proposed method is susceptible to this incomplete and noisy supervisory signal and still able to produce full density depth results. The quantitative results of depth are shown in Table 6. Compared with multi-task learning [35] and unsup-stereo-depthGAN [45] method, our SSDNet multi-task model achieves the best performance thanks to the effective sharing of semantic and boundary features.

**Figure 5:**
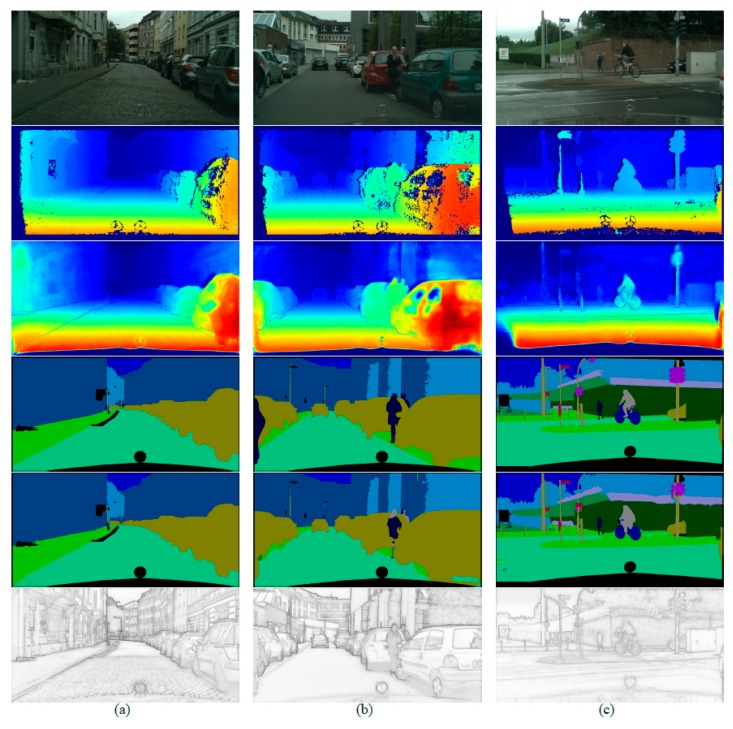
Qualitative results of the SSDNet in the CityScapes dataset. (**a**) and (**b**) show the scenes of large objects, such as vehicles and buildings. (**c**) shows the scene of small objects, such as riders and traffic signs. Top-down order for each column: RGB color image, depth (disparity) groundtruth, depth completion prediction, semantic ground truth, semantic prediction and boundary prediction.

Some qualitative results of the proposed method in the Cityscapes dataset are shown in Figure 5. Each column from top to down displays RGB images, depth (disparity) ground truth, depth completion output, semantic ground truth, semantic prediction and boundary detection result, respectively. Despite the noisy depth ground truth, the proposed model can still benefit from triple-task learning and produce satisfying results.

## 4. Conclusions

In this paper, a multi-task network for simultaneous semantic segmentation and depth completion is proposed. With the structure of single-encoder-multi-decoder, the model is capable of learning the enhanced features suitable for the entire task. Boundary constraint is embedded into the two major tasks via multi-scale feature sharing and cross-task joint loss function. The boundary features play a role of the bridge connecting the semantic and depth prediction tasks and strengthen the relationship between them. The boundary associated cross-task joint loss functions are beneficial for each task. The entire network is implemented end-to-end and evaluated on both synthesized and real datasets. The ablative and comparative results show that our multi-task SSDNet model is able to effectively improve the performance of both semantic segmentation and depth completion tasks in a real-time frame rate.

Future work will focus on further improving the task performance by designing a more robust feature fusion mechanism and better network structure. We also plan to test our algorithm in more complex environments.

## Figures and Tables

**Table 1:** Evaluation metrics.

Metrics For Semantic Segmentation	Pixel accuracy	$Acc=(1/N)\sum_{l}n_{ll}$
	Mean pixel accuracy	$mAcc=(1/nc)\sum_{l}\left(n_{ll}/t_{l}\right)$
	Mean Intersection-over-Union of different categories	$mIoU=(1/nc)\sum_{l}(n_{ll}/(t_{l}+\sum_{k}n_{kl}-n_{ll}))$
	Frequency-weighted IoU	$fwIoU=(1/N)\sum_{l}(t_{l}n_{ll}/(t_{l}+\sum_{k}n_{kl}-n_{ll}))$
Metrics For Depth Completion	Root Mean Squared Error	$RMSE=\sqrt{(1/N)\sum_{j}{\left(d_{j}-{\hat{d}}_{j}\right)}^{2}}$
	Mean Absolute Error	$MAE=(1/N)\sum_{j}\left|d_{j}-{\hat{d}}_{j}\right|$

**Table 2:** Semantic segmentation ablation experiment on Virtual KITTI dataset.

	Acc (%)	mAcc (%)	mIoU (%)	fwIoU (%)	Params	FLOPs
FCN8S [5]	73.549	52.243	28.627	61.948	1.3 × 10^8^	2.2 × 10^10^
BaCNN [20] (baseline)	75.439	54.187	35.190	66.027	1.5 × 10^8^	3.8 × 10^10^
SSDNet_Sem	75.759	58.091	41.786	64.018	2.5 × 10^7^	2.5 × 10^10^
SSDNet_ind	76.427	58.579	41.134	64.823	3.5 × 10^7^	3.3 × 10^10^
SSDNet (our method)	**78.967**	**63.461**	**46.041**	**68.227**	3.5 × 10^7^	3.3 × 10^10^

**Table 3:** Depth Completion Ablation Experiment on Virtual KITTI Dataset.

	0–20 m (cm)		0–50 m (cm)		0–100 m (cm)		Params	FLOPs
	MAE	RMSE	MAE	RMSE	MAE	RMSE		
MRF [39]	56.67	116.776	131.03	312.41	209.45	575.20	n/a	n/a
TGV [40]	41.85	114.57	113.38	323.97	205.78	621.48	n/a	n/a
Sparse-to-dense [25]	258.98	386.91	653.54	1066.55	1072.52	1892.04	n/a	n/a
SparseConvNet [12]	56.44	137.34	153.01	384.96	258.23	681.13	n/a	n/a
SSDNet_Dep	21.97	86.32	87.70	282.63	145.90	473.84	2.5 × 10^7^	2.5 × 10^10^
SSDNet_Dep_d	34.44	113.37	117.59	370.50	169.76	588.63	2.5 × 10^7^	2.5 × 10^10^
SSDNet_Dep_rgb	72.48	199.15	288.73	702.71	455.76	1125.47	2.5 × 10^7^	2.5 × 10^10^
SSDNet_ind	24.96	95.23	86.71	277.04	119.66	404.70	3.5 × 10^7^	3.3 × 10^10^
SSDNet (our method)	**21.65**	**85.05**	**86.47**	**274.03**	**118.58**	**395.51**	3.5 × 10^7^	3.3 × 10^10^

**Table 4:** Semantic segmentation results on CityScapes dataset with a resolution of 512 × 1024 px.

	IoU_cat (%)	IoU_cla (%)	fwt (s)
FCN8S [5]	81.6	61.9	0.5
BaCNN [20] (baseline)	85.6	64.8	0.012
SSDNet (our method)	**86.0**	**65.3**	0.010
ENet [41]	80.4	58.3	0.013
ENet_LSloss [42]	83.6	63.1	0.013
ESPNet [43]	82.2	60.3	0.009
Fast-SCNN [44]	80.5	62.8	0.004
ERFNet [16]	86.5	68.0	0.02
Deeplab LargeFOV [8]	81.2	63.1	4.0

**Table 5:** Detailed IoU (in %) on CityScapes with an image resolution of 512 × 1024 px.

Label_Category	Flat	Nature	Object	Sky	Construction	Human	Vehicle	mIoU
BaCNN [20] (baseline)	97.9	90.8	63.7	92.3	89.6	75.3	89.7	85.6
SSDNet	98.0	91.1	64.6	93.9	90.4	73.1	90.7	86.0

**Table 6:** Depth completion results on CityScapes dataset.

	MAE (px)	RMSE (px)
Multi-task Learning [35]	2.92	5.88
unsup-stereo-depthGAN [45]	n/a	5.44
SSDNet (our method)	**2.85**	**4.44**

## References

[1] Redmon J., Divvala S., Girshick R., Farhadi A. You only look once: Unified, real-time object detection. Proceedings of the IEEE Conference on Computer Vision and Pattern Recognition.

[2] Lin T.Y., Dollár P., Girshick R., He K., Hariharan B., Belongie S. Feature pyramid networks for object detection. Proceedings of the IEEE Conference on Computer Vision and Pattern Recognition.

[3] Girshick R., Donahue J., Darrell T., Malik J. Rich feature hierarchies for accurate object detection and semantic segmentation. Proceedings of the IEEE Conference on Computer Vision and Pattern Recognition.

[4] Ladický L.U., Russell C., Kohli P., Torr P.H. Associative hierarchical crfs for object class image segmentation. Proceedings of the 2009 IEEE 12th International Conference on Computer Vision.

[5] Long J., Shelhamer E., Darrell T. Fully convolutional networks for semantic segmentation. Proceedings of the IEEE Conference on Computer Vision and Pattern Recognition.

[6] Noh H., Hong S., Han B. Learning deconvolution network for semantic segmentation. Proceedings of the IEEE International Conference on Computer Vision.

[7] Badrinarayanan V., Kendall A., Cipolla R. (2017). Segnet: A deep convolutional encoder-decoder architecture for image segmentation. IEEE Trans. Pattern Anal. Mach. Intell..

[8] Chen L., Papandreou G., Kokkinos I., Murphy K., Yuille A.L. (2014). Semantic Image Segmentation with Deep Convolutional Nets and Fully Connected CRFs. arXiv.

[9] Torralba A., Oliva A. (2002). Depth estimation from image structure. IEEE Trans. Pattern Anal. Mach. Intell..

[10] Liu B., Gould S., Koller D. Single image depth estimation from predicted semantic labels. Proceedings of the 2010 IEEE Computer Society Conference on Computer Vision and Pattern Recognition.

[11] Hua J., Gong X. A Normalized Convolutional Neural Network for Guided Sparse Depth Upsampling. Proceedings of the Twenty-Seventh International Joint Conference on Artificial Intelligence.

[12] Uhrig J., Schneider N., Schneider L., Franke U., Brox T., Geiger A. Sparsity invariant cnns. Proceedings of the 2017 International Conference on 3D Vision (3DV).

[13] Simonyan K., Zisserman A. (2014). Very deep convolutional networks for large-scale image recognition. arXiv.

[14] Chen L.C., Papandreou G., Kokkinos I., Murphy K., Yuille A.L. (2017). Deeplab: Semantic image segmentation with deep convolutional nets, atrous convolution, and fully connected crfs. IEEE Trans. Pattern Anal. Mach. Intell..

[15] He K., Zhang X., Ren S., Sun J. Deep residual learning for image recognition. Proceedings of the IEEE Conference on Computer Vision and Pattern Recognition.

[16] Romera E., Alvarez J.M., Bergasa L.M., Arroyo R. (2017). Erfnet: Efficient residual factorized convnet for real-time semantic segmentation. IEEE Trans. Intell. Trans. Syst..

[17] Couprie C., Farabet C., Najman L., LeCun Y. (2013). Indoor semantic segmentation using depth information. arXiv.

[18] Gupta S., Girshick R., Arbeláez P., Malik J. (2014). Learning Rich Features from RGB-D Images for Object Detection and Segmentation. European Conference on Computer Vision.

[19] Wang W., Neumann U. Depth-aware CNN for RGB-D segmentation. Proceedings of the European Conference on Computer Vision (ECCV).

[20] Zou N., Xiang Z., Chen Y., Chen S., Qiao C. (2019). Boundary-Aware CNN for Semantic Segmentation. IEEE Access.

[21] Eigen D., Puhrsch C., Fergus R. (2014). Depth map prediction from a single image using a multi-scale deep network. Proceedings of the 27th International Conference on Neural Information Processing Systems—Volume 2 (NIPS’14).

[22] Eigen D., Fergus R. Predicting depth, surface normals and semantic labels with a common multi-scale convolutional architecture. Proceedings of the IEEE International Conference on Computer Vision.

[23] Ku J., Harakeh A., Waslander S.L. In defense of classical image processing: Fast depth completion on the cpu. Proceedings of the 2018 15th Conference on Computer and Robot Vision(CRV).

[24] Hazirbas C., Ma L., Domokos C., Cremers D. (2016). Fusenet: Incorporating Depth into Semantic Segmentation via Fusion-Based CNN Architecture. Asian Conference on Computer Vision.

[25] Mal F., Karaman S. Sparse-to-dense: Depth prediction from sparse depth samples and a single image. Proceedings of the 2018 IEEE International Conference on Robotics and Automation (ICRA).

[26] Jaritz M., De Charette R., Wirbel E., Perrotton X., Nashashibi F. Sparse and dense data with cnns: Depth completion and semantic segmentation. Proceedings of the 2018 International Conference on 3D Vision (3DV).

[27] Zoph B., Vasudevan V., Shlens J., Le Q.V. Learning transferable architectures for scalable image recognition. Proceedings of the IEEE Conference on Computer Vision and Pattern Recognition.

[28] Zhang Y., Funkhouser T. Deep depth completion of a single RGB-D image. Proceedings of the IEEE Conference on Computer Vision and Pattern Recognition.

[29] Qiu J., Cui Z., Zhang Y., Zhang X., Liu S., Zeng B., Pollefeys M. Deeplidar: Deep surface normal guided depth prediction for outdoor scene from sparse lidar data and single color image. Proceedings of the IEEE Conference on Computer Vision and Pattern Recognition.

[30] Murphy K.P., Torralba A., Freeman W.T. (2004). Using the forest to see the trees: A graphical model relating features, objects, and scenes. Advances in Neural Information Processing Systems.

[31] Teichmann M., Weber M., Zoellner M., Cipolla R., Urtasun R. Multinet: Real-time joint semantic reasoning for autonomous driving. Proceedings of the 2018 IEEE Intelligent Vehicles Symposium (IV).

[32] Sermanet P., Eigen D., Zhang X., Mathieu M., Fergus R., LeCun Y. (2013). Overfeat: Integrated recognition, localization and detection using convolutional networks. arXiv.

[33] Misra I., Shrivastava A., Gupta A., Hebert M. Cross-stitch networks for multi-task learning. Proceedings of the IEEE Conference on Computer Vision and Pattern Recognition.

[34] Uhrig J., Cordts M., Franke U., Brox T. (2016). Pixel-level Encoding and Depth Layering for Instance-Level Semantic Labeling. German Conference on Pattern Recognition.

[35] Kendall A., Gal Y., Cipolla R. Multi-task learning using uncertainty to weigh losses for scene geometry and semantics. Proceedings of the IEEE Conference on Computer Vision and Pattern Recognition.

[36] Gaidon A., Wang Q., Cabon Y., Vig E. Virtual Worlds as proxy for multi-object tracking analysis. Proceedings of the IEEE Conference on Computer Vision and Pattern Recognition.

[37] Cordts M., Omran M., Ramos S., Rehfeld T., Enzweiler M., Benenson R., Franke U., Roth S., Schiele B. The cityscapes dataset for semantic urban scene understanding. Proceedings of the IEEE Conference on Computer Vision and Pattern Recognition.

[38] Wang Z., Bovik A.C., Sheikh H.R., Simoncelli E.P. (2004). Image quality assessment: From error visibility to structural similarity. IEEE Trans. Image Process..

[39] Harrison A., Newman P. (2010). Image and Sparse Laser Fusion for Dense Scene Reconstruction. Field and Service Robotics.

[40] Ferstl D., Reinbacher C., Ranftl R., Rüther M., Bischof H. Image guided depth upsampling using anisotropic total generalized variation. Proceedings of the IEEE International Conference on Computer Vision.

[41] Paszke A., Chaurasia A., Kim S., Culurciello E. (2016). Enet: A deep neural network architecture for real-time semantic segmentation. arXiv.

[42] Berman M., Rannen Triki A., Blaschko M.B. The Lovász-Softmax loss: A tractable surrogate for the optimization of the intersection-over-union measure in neural networks. Proceedings of the IEEE Conference on Computer Vision and Pattern Recognition.

[43] Mehta S., Rastegari M., Caspi A., Shapiro L., Hajishirzi H. Espnet: Efficient spatial pyramid of dilated convolutions for semantic segmentation. Proceedings of the European Conference on Computer Vision (ECCV).

[44] Poudel R.P., Liwicki S., Cipolla R. (2019). Fast-SCNN: Fast semantic segmentation network. arXiv.

[45] Pilzer A., Xu D., Puscas M., Ricci E., Sebe N. Unsupervised adversarial depth estimation using cycled generative networks. Proceedings of the 2018 International Conference on 3D Vision (3DV).

